# Magnetic resonance imaging organ at risk delineation for nasopharyngeal radiotherapy: Measuring the effectiveness of an educational intervention

**DOI:** 10.1002/jmrs.651

**Published:** 2023-02-07

**Authors:** Olivia Ryan, Kylie Dundas, Yolanda Surjan, Doaa Elwadia, Kimberley Nguyen, Michael Cardoso, Shivani Kumar

**Affiliations:** ^1^ College of Health, Medicine and Wellbeing, School of Health Sciences The University of Newcastle Callaghan New South Wales Australia; ^2^ Liverpool and Macarthur Cancer Therapy Centres South Western Sydney Local Health District Liverpool New South Wales Australia; ^3^ Ingham Institute of Applied Medical Research Liverpool New South Wales Australia; ^4^ South Western Sydney Clinical School, Faculty of Medicine University of New South Wales Sydney New South Wales Australia; ^5^ Faculty of Medicine Western Sydney University Sydney New South Wales Australia; ^6^ Centre for Medical Radiation Physics University of Wollongong Wollongong New South Wales Australia

**Keywords:** education, inter‐observer variability, MRI, nasopharyngeal carcinoma, OAR delineation

## Abstract

**Introduction:**

Magnetic resonance imaging (MRI) demonstrates superior soft tissue contrast and is increasingly being used in radiotherapy planning. This study evaluated the impact of an education workshop in minimising inter‐observer variation (IOV) for nasopharyngeal organs at risk (OAR) delineation on MRI.

**Methods:**

Ten observers delineated 14 OARs on 4 retrospective nasopharyngeal MRI data sets. Standard contouring guidelines were provided pre‐workshop. Following an education workshop on MRI OAR delineation, observers blinded to their original contours repeated the 14 OAR delineations. For comparison, reference volumes were delineated by two head and neck radiation oncologists. IOV was evaluated using dice similarity coefficient (DSC), Hausdorff distance (HD) and relative volume. Location of largest deviations was evaluated with centroid values. Observer confidence pre‐ and post‐workshop was also recorded using a 6‐point Likert scale. The workshop was deemed beneficial for an OAR if ≥50% of observers mean scores improved in any metric and ≥50% of observers' confidence improved.

**Results:**

All OARs had ≥50% of observers improve in at least one metric. Base of tongue, larynx, spinal cord and right temporal lobe were the only OARs achieving a mean DSC score of ≥0.7. Base of tongue, left and right lacrimal glands, larynx, left optic nerve and right parotid gland all exhibited statistically significant HD improvements post‐workshop (*P* < 0.05). Brainstem and left and right temporal lobes all had statistically significant relative volume improvements post‐workshop (*P* < 0.05). Post‐workshop observer confidence improvement was observed for all OARs (*P* < 0.001).

**Conclusions:**

The educational workshop reduced IOV and improved observers' confidence when delineating nasopharyngeal OARs on MRI.

## Graphical abstract

Magnetic resonance imaging demonstrates superior soft tissue contrast and is increasingly being used in radiotherapy planning. This study evaluated the impact of an education workshop in minimising inter‐observer variation for nasopharyngeal organ at risk delineation on MRI. Our results found that the educational workshop reduced inter‐observer variation and improved observers' confidence.

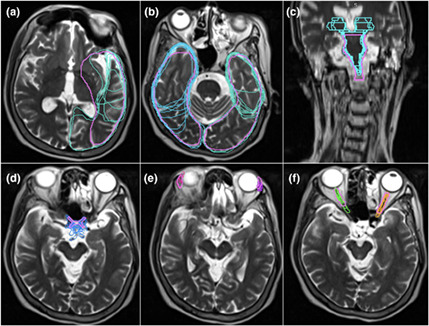

## Introduction

Radiotherapy (RT) for nasopharyngeal carcinoma is constrained by the proximity of organs at risk (OARs) to tumour volumes. Modern RT techniques allow high doses of radiation to be delivered to target volumes while sparing nearby OARs.^1^ This significantly improves local control, whilst reducing treatment‐related side effects, which improves patients' quality of life.^1^ To ensure the dose delivered to the tumour volume is precise and the dose to OARs is within tolerance, accurate delineation of OARs is necessary.^2^ Inter‐observer variability (IOV) contributes to delineation error in RT and impacts on the dose delivered to OARs.^3^, ^4^ IOV is a measure of the difference between contours completed by two or more observers examining the same material.^2^ Measures designed to minimise IOV include guidelines and atlases, multi‐modality imaging, standard protocols and auto‐contouring tools.^2^

The inclusion of Magnetic resonance imaging (MRI) in nasopharyngeal RT has shown reductions in IOV.^5^ Compared to computed tomography (CT), MRI provides superior soft‐tissue visualisation, which improves target volume and OAR delineation.^6^ Due to the complexity of soft‐tissue structures in the head and neck (H&N) region, MRI improves nasopharyngeal OAR delineation.^7^ These OARs display similar soft‐tissue contrast to surrounding structures on CT data sets but are more discernible on MRI scans and thus better delineated.^8^, ^9^, ^10^, ^11^ MRI is often combined with CT to optimise delineation of target volumes and OARs while maintaining accurate dose calculation.^12^ Currently, the existing CT‐based treatment planning workflow relies on target and OAR definition on MRI and a transfer of contours to CT via image registration.^12^ MRI‐CT co‐registration requires two separate imaging sessions and has some fundamental and logistical drawbacks as dual‐modality workflows may introduce misregistration and geometrical uncertainties.^12^ MRI‐only workflows are feasible; however, radiation therapists (RTs) need to be educated on how to identify and delineate OARs on MRI as well.^12^

Education courses conducted in‐person, online or a combination of both, have shown to be effective teaching modalities.^13^, ^14^, ^15^ Davis et al.^13^ demonstrated didactic and hands‐on interventions were more effective in facilitating change than didactic sessions alone. In this meta‐analysis^13^ studies with only didactic interventions had no statistically significant impact on participants' behaviour or healthcare outcomes, while studies with both didactic sessions and interactive interventions did. In a study by Awan et al.,^14^ seven resident observers contoured 26 H&N OARs on a CT scan. After a teaching intervention, the observers contoured the same 26 OARs on another CT scan. The teaching intervention involved an atlas and real‐time software‐based feedback to help contour OARs in the H&N region. The mean DSC scores across all structures improved between phases, and each resident observer demonstrated statistically significant improvements in overall OAR contouring (*P* < 0.01). These findings indicate educational interventions can improve the contouring of H&N OARs. As a result of these studies, we explored the possibility of using an interactive approach, so a didactic lesson on radiological anatomy in an interactive environment was used. The lack of published clinical findings on the effects of a teaching intervention for MRI‐only nasopharyngeal OAR delineation, emphasise the need for this research.^2^

This study evaluated whether participation in an education workshop minimises IOV when delineating nasopharyngeal OARs on MRI only.

## Methodology

### Context

The South Western Sydney Local Health District Human Research Ethics Committee approved this study (HREC/16/LPOOL/603), which utilised retrospective imaging data obtained during routine clinical radiotherapy planning. Retrospective MRI data sets of five patients diagnosed with nasopharyngeal carcinoma were used. Inclusion criteria were patients treated for nasopharyngeal carcinoma, with a curative treatment intent, prescribed dose ≥60Gy, with any tumour and nodal stage but no metastasis present and had MRI scans including the temporal lobe superiorly and clavicles inferiorly.

This study was a single‐centre, comparison study of OAR IOV pre and post a contouring education workshop intervention. Eleven radiation therapist observers were asked to contour 14 H&N OARs on five patient MRI data sets pre and post the education workshop. The 14 OARs delineated included: base of tongue, brainstem, left and right lacrimal glands, larynx, optic chiasm, left and right optic nerves, left and right parotid glands, pharyngeal constrictors, spinal cord and left and right temporal lobes. These OARs were selected as they display similar soft tissue contrast to surrounding structures on a CT data set but are more discernible on MRI scans.

### Imaging

Images were acquired on a 3 T MRI scanner (Magnetom Skyra; Siemens Healthcare, Erlangen, Germany) with two 18‐channel receiver array coils. The surface coils were placed over the H&N region abutting each other using coil bridges on top of a H&N mask to cover the area of interest. Images were acquired in the transverse plane using T2_tse_DIXON sequence. Dixon in‐phase images were utilised for delineation in this study. In‐phase images are a combination of water and fat. The voxel size was 1x1x3mm, slice thickness was 3 mm with a 0‐slice gap, base resolution was 256, and the flip angle was 140°. The 250 mm field of view extended from the superior aspect of the orbital bone to the suprasternal notch. The repetition time was 1459.0 ms, while echo time was 83.0 ms.

### Pre‐workshop delineation

Before the workshop, observers delineated 14 OARs using MIM version 6.9.5 (MIM software inc., Cleveland, Ohio). The observers were provided with H&N contouring work instructions based on consensus delineation guidelines for H&N OARs.^8^ The instructions described the borders of each OAR and an example image of the OAR contoured on a CT scan. The observers were blinded to each other's volumes. Observers were asked to indicate their experience levels as either no previous experience (never rostered for head/neck planning), some experience (≤3 months of consistently contouring a minimum of 5 plans a week) or experienced (≥6 months of consistently contouring a minimum of 5 plans on week). Observers were also asked to rank their levels of confidence when contouring each OAR on a 6‐point Likert scale ranging from 0–5 indicating not confident, slightly confident, somewhat confident, fairly confident, confident and very confident.

### Intervention

The education workshop was conducted after observers finalised their initial volumes. The workshop included discussion on basic concepts of T1 and T2 weighted MRI scans, OAR anatomy in relation to other structures and their typical appearance on T2_DIXON_in‐phase acquisition, and how to recognise structures on this acquisition such as water, fat and bone. The 14 OARs were discussed in detail on the appearance of each OAR on T2_DIXON In‐phase sequence with images for aid. The education workshop was collaboratively developed by a senior MRI radiographer with more than 12 years of MRI experience employed in a radiotherapy department and a senior radiation therapist with expertise in H&N planning and MRI guided radiotherapy. As COVID‐19 restrictions prohibited in‐person workshops, the education intervention was held virtually. Observers were encouraged to ask questions and received real‐time feedback.

### Post‐workshop delineation

To minimise recall bias, all observers were given a four‐week break between pre‐ and post‐workshop delineations, data sets were re‐labelled, and observers were blinded to their pre‐workshop volumes. Observers were given the same instructions as pre‐workshop along with new information from the education workshop. Observers were once again asked to rank their confidence levels for each OAR following delineations. In addition, observers completed a 5‐point Likert scale survey indicating whether they strongly disagreed, disagreed, were neutral, agreed or strongly agreed with being comfortable with H&N OAR contouring pre‐ and post‐workshop, and whether the education workshop was a worthwhile experience.

### Reference volumes

Reference volumes were generated based on the consensus of two H&N radiation oncologists (RO) with extensive experience in utilising MRI for H&N radiotherapy. Each RO has contoured between 40–50 cases over the last 5 years. Their contours have been routinely audited weekly by other H&N RO's and radiologists at the hospital. For this study, each RO contoured seven OARs and then audited the other seven. If a contour was not accepted by the auditing RO, edits were made after discussion and agreement. The ROs followed international consensus guidelines to aid with contouring.^8^

### Analysis

Contour analysis was performed in MIM version 6.9.5 (MIM software inc., Cleveland, Ohio). The data obtained for each OAR included DSC, HD (mm), absolute volume (cc) and centroid X, Y and Z (cm). The relative volume (%) and centroid difference (Δ) between each observer's OAR and the reference OAR were reported. DSC measures deviations of observers' contours from reference contours and evaluates their overlap.^4^ A DSC ≥0.7 indicated a ‘good’ agreement between observer and reference contours.^2^ HD identifies the greatest distance of a point in one contour to the closest point in another contour.^4^ A high HD indicates greater dissimilarity between the observers' contour and reference contour, while a HD of zero indicates identical contours.^4^ Relative volume is a volumetric comparison between observer and reference volumes.

To calculate relative volume:

$$\text{Relative volume}\left(\%\right)=\begin{array}{l} \frac{\text{Observers absolute volume}\left(\mathrm{cc}\right)}{\text{Reference absolute volume}\left(\mathrm{cc}\right)}\\ \times 100. \end{array}$$

Centroid ΔX, ΔY and ΔZ describe the difference in geometric centre of a volume in 3 planes, compared to the reference volume. The X‐axis represents left/right, Y‐axis represents anterior/posterior, and Z‐axis represents superior/inferior.

To calculate centroid ΔX:

CentroidΔX=observers centroidX−reference centroidX

The same equation was followed to calculate centroid ΔY and ΔZ.

Quantitative analysis of pre‐ and post‐workshop differences involved DSC, HD, relative volume, centroid ΔX, ΔY and ΔZ and confidence levels for each OAR for all observers being analysed. Statistical analysis was performed using the Mann–Whitney U test on the program SPSS (IBM Corp. Released 2021. IBM SPSS Statistics for Macintosh, Version 28.0. Armonk, NY: IBM Corp), a *P*‐value of < 0.05 was considered significant.

### Analysis of Education Program

The education workshop was deemed beneficial for any OAR if ≥50% of observer contours demonstrated improvement based on IOV comparison metrics and if ≥50% of observer confidence improved.

## Results

The study was conducted from May to August 2021. We began with 11 observers and five patient data sets; however, due to a COVID‐19 outbreak putting extra strain on staff, one observer dropped out and one patient data set was removed due to time constraints. COVID‐19 restrictions prohibited face‐to‐face workshops, so the education intervention had to be presented virtually. Observers experience in H&N radiotherapy varied, 4 of 10 observers had no previous experience, three had some experience, and 3 observers were experienced. The four data sets used were of patients with the following staging: T4N2M0, T2N2M0, T1N2M0 and T3N3bM0.

As shown in Table 1, all OARs except the right optic nerve, pharyngeal constrictors and spinal cord had statistically significant improvements in at least one of the metrics measuring inter‐observer variation. Interestingly, the left optic nerve and right parotid gland demonstrated statistically significant improvement for HD across observers; however, the bilateral structures did not. All OARs had statistically significant improvements in observers' confidence levels when contouring each OAR.

**Table 1:** The mean, *P*‐values and *u*‐values for each organ at risk (OAR) for dice similarity coefficient (DSC), Hausdorff distance (HD), relative volume and centroid ΔX, ΔY and ΔZ and the mode, *P*‐values and *u*‐values for each OARs confidence level

OAR	Timepoint	DSC			HD (mm)			Relative volume (%)			Centroid ΔX (cm)			Centroid ΔY (cm)			Centroid ΔZ (cm)			Confidence Level		
		Mean (SD)	*P*‐value	*u*‐value	Mean (SD)	*P*‐value	*u*‐value	Mean (SD)	*P*‐value	*u*‐value	Mean (SD)	*P*‐value	*u*‐value	Mean (SD)	*P*‐value	*u*‐value	Mean (SD)	*P*‐value	*u*‐value	Mode	*P*‐value	*u*‐value
Base of tongue	Pre‐Workshop	0.6 (0.1)	0.13*	1059.0	12.5 (4.5)	0.007*	520.0	99.0 (48.9)	0.252	919.0	0.0 (0.1)	0.295	691.5	0.2 (0.3)	0.091	624.5	0.0 (0.6)	0.172	942.0	2	<0.001*	1345.5
	Post‐Workshop	0.7 (0.1)			9.8 (3.6)			103.0 (34.6)			0.0 (0.1)			0.1 (0.2)			0.1 (0.5)			4		
Brainstem	Pre‐Workshop	0.8 (0.1)	0.513	732.0	11.6 (8.2)	0.507	869.0	83.3 (21.1)	0.006*	1087.0	0.0 (0.1)	0.069	611.0	−0.1 (0.1)	0.521	733.5	0.4 (0.3)	0.464	876.0	4	<0.001*	1145.5
	Post‐Workshop	0.7 (0.1)			12.9 (7.5)			105.1 (36.2)			−0.1 (0.1)			−0.1 (0.1)			0.6 (0.5)			5		
Lacrimal gland left	Pre‐Workshop	0.2 (0.2)	0.104	968.0	12.9 (3.6)	<0.001*	445.0	56.2 (46.4)	0.851	780.5	0.2 (0.2)	0.045*	592.0	0.2 (0.3)	0.560	860.5	−0.7 (0.5)	<0.001*	1169	2	<0.001*	1265.5
	Post‐Workshop	0.2 (0.1)			10.4 (3.0)			54.6 (41.9)			0.1 (0.2)			0.2 (0.3)			−0.3 (0.5)			5		
Lacrimal gland right	Pre‐Workshop	0.2 (0.2)	0.281	911.5	13.6 (5.5)	<0.001*	443.5	47.6 (30.0)	0.444	879.5	−0.2 (0.2)	0.025*	1033.5	0.2 (0.3)	0.532	735.0	−0.7 (0.7)	0.014*	1055.5	2	<0.001*	1265.5
	Post‐Workshop	0.3 (0.2)			9.3 (3.1)			54.6 (34.3)			−0.1 (0.2)			0.1 (0.2)			−0.3 (0.4)			5		
Larynx	Pre‐Workshop	0.6 (0.1)	0.008*	1077.0	17.4 (5.7)	<0.001*	377.5	154.6 (58.4)	0.119	638.0	0.0 (0.0)	0.253	918.5	0.1 (0.2)	0.187	937.0	−0.4 (0.7)	0.046*	1007.0	3	<0.001*	1186.5
	Post‐Workshop	0.7 (0.0)			12.6 (5.2)			135.3 (39.4)			0.0 (0.0)			0.1 (0.1)			−0.1 (0.6)			4		
Optic chiasm	Pre‐Workshop	0.2 (0.2)	0.742	813.5	11.6 (3.1)	0.969	776.0	139.3 (162.1)	0.249	662.5	0.0 (0.1)	0.441	858.5	0.5 (0.3)	0.043*	573.5	−0.3 (0.4)	0.099	948.0	2	<0.001*	1250.0
	Post‐Workshop	0.2 (0.2)			11.2 (2.8)			92.4 (65.8)			0.0 (0.1)			0.3 (0.4)			−0.1 (0.4)			3		
Optic nerve left	Pre‐Workshop	0.4 (0.1)	0.802	826.0	11.8 (3.0)	0.003*	496.5	35.1 (19.3)	0.700	760.0	−0.1 (0.2)	0.405	886.5	0.1 (0.3)	0.722	763.0	0.0 (0.2)	0.051	1003.0	4	<0.001*	1144.5
	Post‐Workshop	0.4 (0.1)			9.9 (1.7)			32.6 (5.3)			−0.1 (0.1)			0.0 (0.3)			0.1 (0.2)			5		
Optic nerve right	Pre‐Workshop	0.4 (0.1)	0.266	915.5	11.9 (4.0)	0.298	692.0	34.4 (17.4)	0.651	753.0	0.1 (0.2)	0.780	771.0	0.1 (0.3)	0.836	778.5	−0.2 (0.2)	0.111	965.5	4	<0.001*	1144.5
	Post‐Workshop	0.4 (0.1)			10.0 (1.2)			32.6 (13.2)			0.1 (0.2)			0.1 (0.3)			−0.2 (0.2)			5		
Parotid Gland left	Pre‐Workshop	0.7 (0.1)	0.152	949.0	15.6 (5.9)	0.201	667.0	58.1 (17.4)	0.052	1002.0	0.3 (0.2)	<0.001*	431.5	0.1 (0.1)	0.067	990.5	0.0 (0.3)	0.019*	1043.5	3	<0.001*	1210.0
	Post‐Workshop	0.7 (0.1)			13.6 (4.2)			66.9 (18.1)			0.1 (0.1)			0.2 (0.1)			0.2 (0.3)			5		
Parotid Gland right	Pre‐Workshop	0.7 (0.1)	0.413	885.0	17.3 (5.7)	0.041*	588.0	56.3 (17.1)	0.419	884.0	−0.3 (0.2)	0.132	956.5	0.1 (0.1)	0.640	848.5	0.0 (0.3)	0.074	985.5	3	<0.001*	1227.5
	Post‐Workshop	0.7 (0.1)			14.6 (5.0)			61.0 (18.7)			−0.3 (0.2)			0.1 (0.1)			0.1 (0.2)			5		
Pharyngeal constrictor	Pre‐Workshop	0.5 (0.1)	0.089	623.0	24.3 (16.0)	0.744	766.0	24.3 (16.0)	0.453	722.0	0.0 (0.1)	0.904	787.5	−0.1 (0.4)	0.220	927.5	0.5 (1.7)	0.939	792.0	2	<0.001*	1465.5
	Post‐Workshop	0.5 (0.2)			20.0 (8.2)			20.0 (8.2)			0.0 (0.1)			0.0 (0.2)			0.2 (1.1)			3		
Spinal cord	Pre‐Workshop	0.7 (0.1)	0.059	996.0	9.8 (9.2)	0.411	885.5	69.6 (26.6)	0.679	843.0	0.0 (0.0)	0.988	801.5	0.0 (0.2)	0.350	897.0	0.4 (1.0)	0.081	618.5	4	<0.001*	1136.5
	Post‐Workshop	0.7 (0.1)			9.2 (6.1)			69.2 (22.6)			0.0 (0.1)			0.0 (0.1)			0.0 (0.7)			5		
Temporal lobe left	Pre‐Workshop	0.5 (0.1)	<0.001*	1163.0	41.4 (9.2)	0.676	843.5	46.0 (19.9)	0.005*	1091.0	0.3 (0.2)	0.068	989.5	−1.7 (0.7)	0.030*	1026.0	−0.6 (0.5)	<0.001*	1195.0	2	<0.001*	1308.0
	Post‐Workshop	0.6 (0.1)			41.3 (12.6)			63.0 (31.5)			0.4 (0.4)			−1.2 (1.0)			−0.2 (0.5)			4		
Temporal lobe right	Pre‐Workshop	0.6 (0.1)	<0.001*	1202.0	37.9 (9.4)	0.900	787.0	49.9 (18.3)	0.001*	1132.0	−0.1 (0.2)	0.018*	555.0	−1.5 (0.7)	0.019*	1044.5	−0.6 (0.4)	<0.001*	1236.5	2	<0.001*	1308.0
	Post‐Workshop	0.7 (0.1)			37.6 (14.0)			66.0 (29.2)			−0.2 (0.2)			−1.1 (0.9)			−0.3 (0.4)			4		

*Note*: * and Bold indicates statistically significant results.

**Figure 1:**
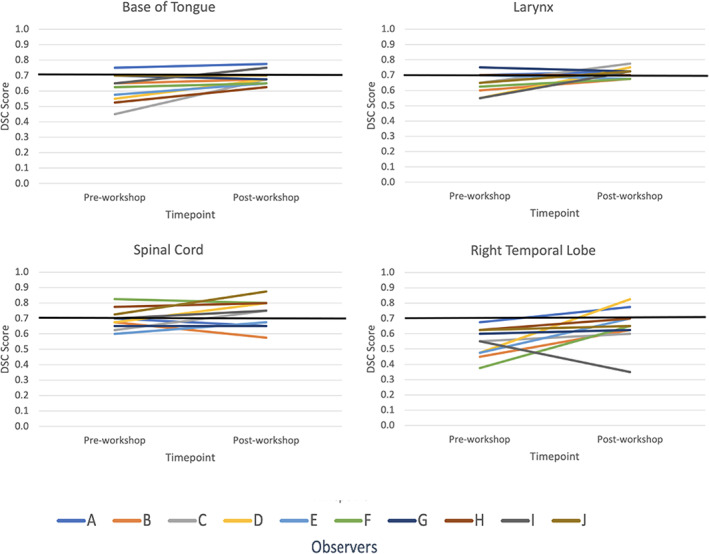
Organs at risk where ≥50% of observers showed an increase in mean dice similarity scores and achieved a mean DSC ≥0.7. DSC, dice similarity coefficient (DSC ≥0.7 indicates ‘good’ agreement).

**Figure 2:**
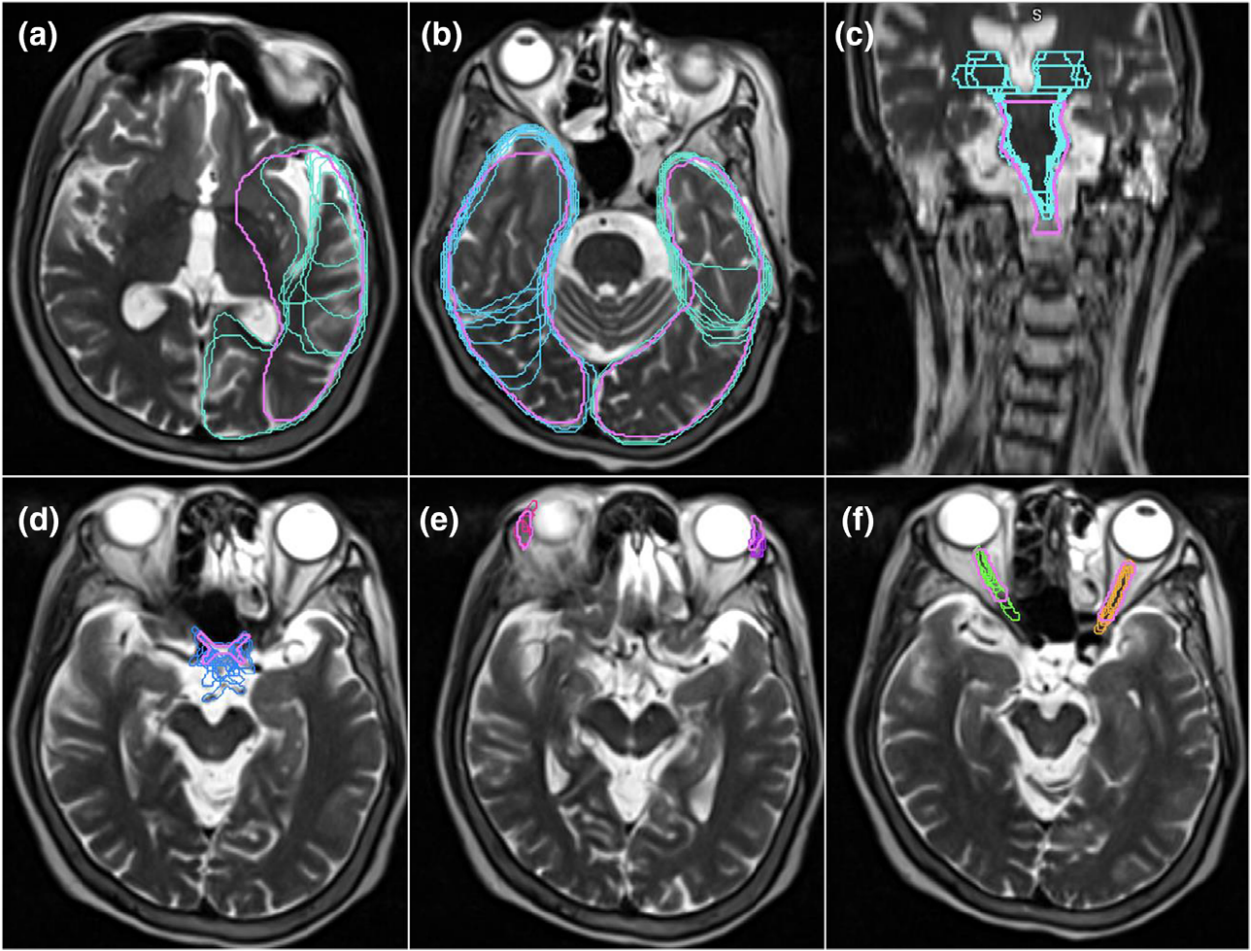
Variation in observer volumes post‐workshop. Screenshots of post‐workshop observer contours from MRI data sets in our study (pink contours indicate the radiation oncologist contoured reference volumes). (a) Left temporal lobe, (b) Left and right temporal lobes, (c) Brainstem, (d) Optic chiasm, (e) Left and right lacrimal glands, (f) Left and right optic nerves.

**Figure 3:**
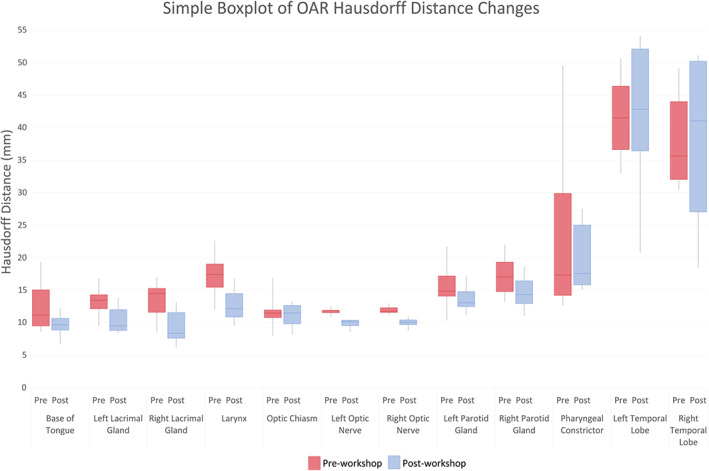
Organs at risk that ≥50% of observers showed an improvement in mean Hausdorff distance scores. OAR = organ at risk, HD = Hausdorff distance (Higher HD indicates more dissimilarity, a HD of 0 indicates the contours contour boundaries match).

Mean DSC scores increased for ≥50% of observers on eight OARs (base of tongue, left and right lacrimal glands, larynx, optic chiasm, spinal cord, left and right temporal lobes). However, only base of tongue, larynx, spinal cord and right temporal lobe also achieved a mean DSC score of ≥0.7. The right and left temporal lobes demonstrated an outlier observer post‐workshop DSC score of 0.4 and 0.3, respectively (Fig. 1 and Figure S1). These outliers, however, had minimal impact on the mean post‐workshop DSC scores. The mean DSC for the right temporal lobe went from 0.66 with the outlier included to 0.69 without it. Similarly, the mean DSC for the left temporal lobe went from 0.62 with the outlier included to 0.66 without. Figure 2A–C are screenshots of an axial slice of observer's post‐workshop optic chiasm, lacrimal glands and optic nerves contours from one of the patient data sets. These structures all had low mean DSC scores of ≤0.4 post‐workshop. As shown in Figure 3, mean HD scores decreased for ≥50% of observers on 12 OARs (base of tongue, left and right lacrimal glands, larynx, optic chiasm, left and right optic nerves, left and right parotid glands, pharyngeal constrictors, left and right temporal lobes). For mean relative volume, ≥50% of observers improved for 10 OARs (base of tongue, brainstem, left lacrimal gland, right lacrimal gland, larynx, optic chiasm, left parotid gland, right parotid gland, left temporal and right temporal lobe). For Centroid ΔX, ΔY and ΔZ, all OARs except left temporal lobe (Fig. 2A) for ΔX axis (left/right), left and right temporal lobes (Fig. 2B) for ΔY axis (anterior/posterior), and brainstem (Fig. 2C) for ΔZ axis (superior/inferior) had a post‐workshop mean centroid difference of ≤0.3 cm. Mean ΔX for left temporal lobe was 0.4 cm, mean ΔY for left and right temporal lobes were −1.2 cm and −1.1 cm, and mean ΔZ for brainstem was 0.6 cm.

**Figure 4:**
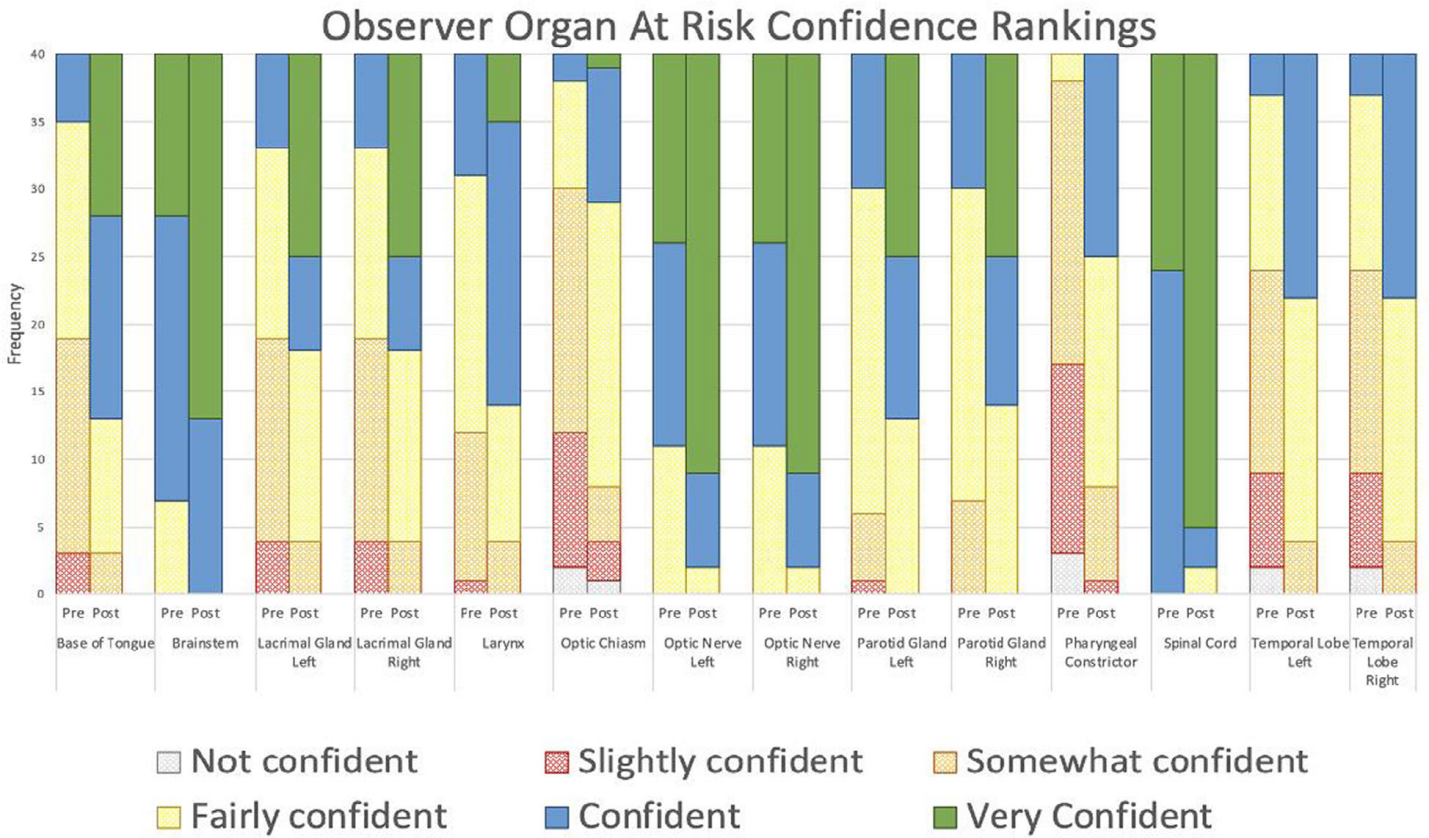
Observers' confidence levels for each organ at risk on the four patient data sets, pre and post the education workshop intervention.

**Figure 5:**
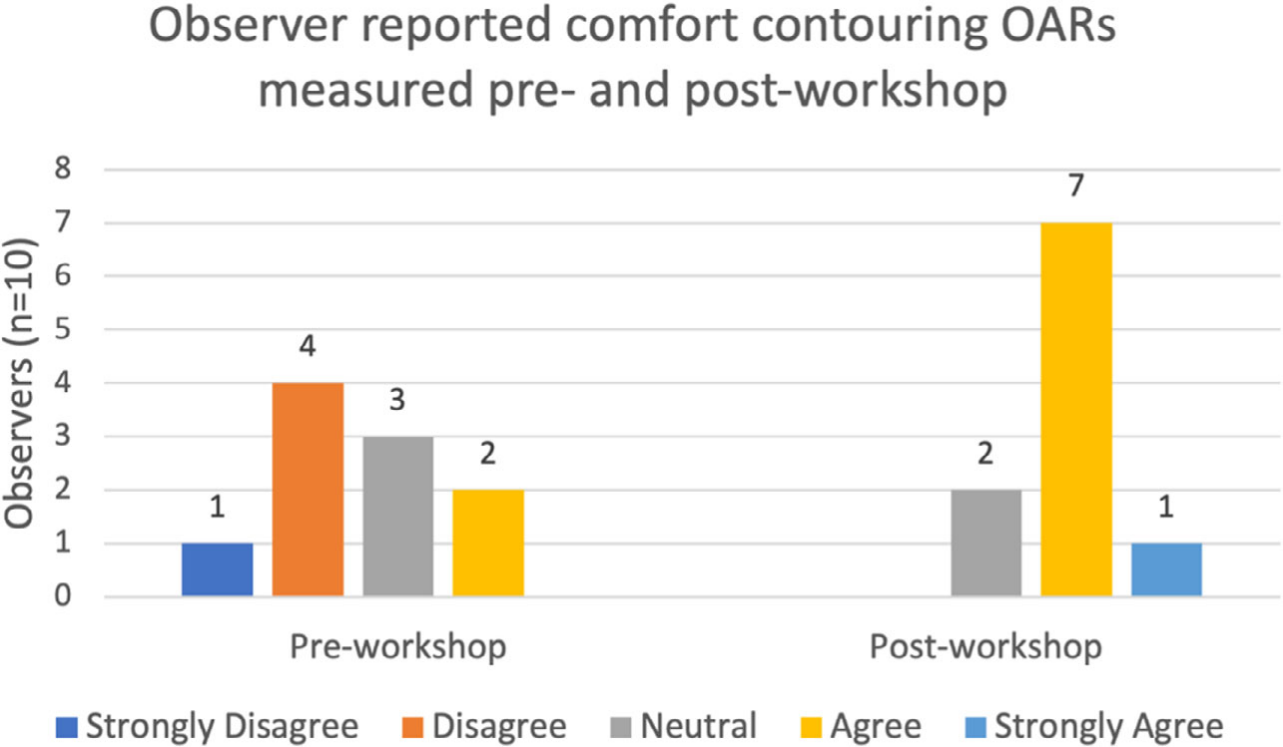
Observer reported comfort with head and neck OAR contouring pre‐ and post‐workshop. OAR, organ at risk.

The percentage of OARs contoured by observers without previous experience had a higher percentage of improvement in mean DSC and relative volume scores. However, the percentage of organs with Hausdorff distance improvements were similar between the groups (Table 2). An increase in confidence was also seen for all 14 OARs post‐workshop (Fig. 4). All observers had an improvement in comfort levels with MRI OAR delineation after the education workshop (Fig. 5). Out of the 10 observers, four observers agreed the education workshop was a worthwhile experience and 6 observers strongly agreed.

**Table 2:** Observer experience and percentage of contoured OARs with IOV improvements

Level of experience	Number of observers (*n* = 10)	Percentage of OARs that exhibited DSC improvements	Percentage of OARs that exhibited HD improvements	Percentage of OARs that exhibited relative volume improvements
No experience, never rostered for H&N planning	4	52%	71%	64%
≤3 months H&N neck planning rotation (some experience)	3	38%	74%	50%
≥6 months H&N planning rotation (experienced)	3	38%	74%	45%

Abbreviations: DSC, dice similarity coefficient; HD, Hausdorff distance; OV, inter‐observer variation; OAR, organ at risk.

## Discussion

Contouring variability is a well‐known source of geometric error in radiotherapy planning.^2^, ^16^ The impact of contour deviation has been associated with poorer clinical outcomes.^2^ RTs are highly skilled in CT image interpretation as CT imaging is routinely used for training and clinically. With the increased use of MRI in radiotherapy, including MRI‐only workflows, it is important RTs are skilled in identifying and delineating OARs on MRIs too. MRI guidelines for radiotherapy H&N contouring do not exist, and therefore, there are no guidelines to specify optimal sequences for OAR delineation. Current international guidelines are CT‐based and only include CT‐MRI fused datasets, signifying a need for future consideration of including MRI‐only data sets in these guidelines.^8^ MRI delineation skills are also valuable as the introduction of automated workflows becomes more routine, and RTs will be required to make a qualitative assessment on whether the generated delineation is correct.^17^ This is a novel study investigating the benefits of an educational workshop on reducing IOV when contouring in the H&N region specifically on MRI. The results showed the education workshop was beneficial in reducing IOV and improving observer confidence when delineating nasopharyngeal OARs on MRI scans.

There have been previous CT‐based studies in this area, and our study supports previous findings that teaching interventions can reduce IOV when contouring H&N OARs.^1^, ^2^, ^14^, ^15^, ^18^, ^19^, ^20^ In the Bekelman et al.^18^ study, 11 radiation oncology residents contoured three H&N clinical tumour volumes (CTV) on CT scans before and after a teaching intervention. Six observers did not have prior experience in H&N contouring, and five observers did. The teaching intervention consisted of a didactic session on identifying anatomic landmarks on cross‐sectional images and a hands‐on practical session on CTV target delineation. Observers' contours were rated as adequate or inadequate, based on current radiotherapy protocols. For the group without prior experience, 60%, 0% and 20% of baseline contours were deemed adequate as compared to 100%, 40% and 80% in follow‐up contours. Likewise, for the group with prior experience, 83%, 33% and 67% of baseline contours were deemed appropriate compared to 100%, 67% and 100% for the follow‐up contours. Similarly, to our study, these findings suggest incorporating a teaching intervention led to improved delineation for participants, but especially for those with no prior experience.

Our pilot study only showed a short‐term assessment on the education impact, with constant utilisation the clinical implication could be different. While observer confidence is a simplistic measure for the impact of the workshop, it's still relevant to know observer perception on the material delivered and if they felt they could identify OARS on MRI. In our study, we demonstrated the education workshop improved observers' contouring confidence levels, as all observers' confidence levels increased for all OARs post‐workshop. An increase in confidence was associated to an improvement in contouring consistency, as all OARs also showed a decrease in IOV. Similarly, Jaswal et al.^20^ demonstrated an increase in confidence scores as well as improvement in contouring, as across all contoured H&N structures there was a 0.20 median improvement in students' average DSC score (*P* < 0.001). Whereas Stanley et al.^21^ found observer confidence was not reflected in contouring consistency as the ratio of smallest to largest contour volumes for each brain metastasis contoured by eight physicians varied from 1.25 to 4.47, indicating a high degree of IOV. The average observer's confidence, on the other hand, was relatively high, with a mean score of 3.2, on a scale where 4 indicated very high confidence.

In our study, observer's level of experience was related to improvements in DSC and relative volume scores, as the percentage of OARs contoured by observers with no previous experience that improved post‐workshop was higher than the percentage of OARs that improved contoured by observers with previous experience. This is similar to other studies^14^, ^18^ reporting that a teaching intervention improved contouring particularly for participants without previous experience. The level of experience of observers did not seem to influence their HD scores as all observers improved at the same rate regardless of level of experience.

Our study also found the largest centroid ΔX and ΔY variations were demonstrated for the temporal lobes (Fig. 2A,B). Since an OAR's volume scales faster than its surface area, the larger centroid ΔX and ΔY variations may be a consequence of the temporal lobe's large volume.^4^ Both the right and left temporal lobes demonstrated outlier DSC scores of 0.4 and 0.3, respectively, post‐workshop (Fig. 1 and Figure S1). A single observer was responsible for both outliers. This observer indicated no previous experience with head/neck planning, so this lack of experience may have contributed to the outliers. The brainstem had the largest centroid ΔZ variation (Fig. 2C) predominantly due to variability in defining the brainstem and spinal cord boundary. Brainstem DSC (Fig. S1) and HD scores were also better before the education workshop. Similarly, spinal cord, optic nerves, parotid glands and pharyngeal constrictors DSC's remained equivalent post‐workshop (Fig. S1). These structures are commonly delineated on CT by RTs, this familiarity with CT‐based delineation may have resulted in better concordance than post‐workshop. However, better concordance does not necessarily mean the contours are accurate. Despite this lack of improvement, the brainstem, spinal cord and parotid glands post‐workshop mean DSC's were 0.7, which is considered a ‘good’ agreement. However, the left and right optic nerve structures had low mean DSC scores of 0.4 post‐workshop and mean HD values of 1 cm post‐workshop (Fig. 2F). This HD value is clinically significant due to the optic nerves small size. Because of their small size and tubular geometry, optic nerves are difficult to delineate. Small absolute differences can also lead to poor scores when the volume of the organ is small.^22^ Our results also showed statistically significant HD improvements for only one bilateral structure for the optic nerve and parotid glands. This was caused by the small sample size and outliers in the data; however, this statistical significance does not indicate clinical significance. The pharyngeal constrictors had a post‐workshop mean DSC score of 0.5. This low DSC score may have been due to RTs CT familiarity. The pharyngeal constrictors are difficult to visualise on CT, so its contouring requires the accurate interpretation of guidelines based on anatomical landmarks.^22^ The observers may have contoured the pharyngeal constrictors based on perceived CT boundaries, which may have contributed to the higher degree of variations observed in this study for MRI. Interestingly, for the optic chiasm (Fig. 2D), post‐workshop DSC scores improved but remained under the 0.7 threshold (Fig. S2). This may have been due to the use of T2_DIXON affecting its visibility, or it may be due to learnt behaviour from CT delineation. Likewise, the lacrimal glands (Fig. 2E) improved across all metrics but still had low DSC scores (Fig. S2). This may have been due to distortion at the edge of the field of view on MRI and possible motion artefacts from eye movement.^22^ The orbital area is prone to distortions and signal loss on MRI scans due to interfaces among air, bone and soft tissue, resulting in an inhomogeneous magnetic field and susceptibility artefacts.^22^

This study had some limitations. The small sample size of observers and patient data sets may have limited the ability to test the effectiveness of the education workshop. Also, COVID‐19 restrictions required the education workshop to be delivered virtually, thereby limiting observer hands‐on and interactive experience. Due to a COVID‐19 outbreak putting additional strain on staff, one observer dropped out and one patient data set was removed from the study due to incomplete observer data. Observers may also have been influenced by CT recall bias, since they may have delineated organs based on perceived CT boundaries. In addition, the reference contours created by consensus of two ROs may have IOV. For future work, incorporating consensus volumes of ROs and radiologists may provide more accurate reference volumes. Contours were also only evaluated quantitatively, not qualitatively, so future studies should incorporate qualitative RO assessments of the clinical significance of volume changes. Further research is also needed to determine the most appropriate imaging sequence for each organ.

## Conclusion

This study demonstrated educational workshops can potentially reduce inter‐observer variability and improve observer confidence when delineating nasopharyngeal OARs on MRI scans. This study provides important insights concerning the emerging trend of MRI‐only radiotherapy planning workflows. It is a pilot study adding to a body of evidence regarding the emerging role of MRI in radiotherapy. All observers found the teaching intervention a valuable experience and all reported improvements in confidence levels post‐workshop. This is consistent with other studies of teaching interventions for contouring in radiation oncology and calls for investment and additional research in this area.

## Funding information

This work was conducted as part of an honours candidature through the University of Newcastle. The primary author was the recipient of a scholarship awarded by the South Western Sydney Local Health District (SWSLHD).

## Conflict of interest

SWSLHD has a research agreement with Siemens Healthineers AG. However, no part of the design or execution of this study was conducted under this research agreement.

## Ethics Statement

This study was approved by the local human research ethics committee.

## Supporting information


**Data S1** Supporting Information

## Data Availability

Research data may be available after seeking appropriate ethical and governance approvals.
