# Supplementary material for: Magnetic resonance imaging organ at risk delineation for nasopharyngeal radiotherapy: Measuring the effectiveness of an educational intervention
Source: J Med Radiat Sci. 2023 Feb 7;70(Suppl 2):59–69. doi: 10.1002/jmrs.651 (PMC10122931; doi:10.1002/jmrs.651)

**Supporting Information**

**Magnetic resonance imaging (MRI) organ at risk delineation for nasopharyngeal radiotherapy: measuring the effectiveness of an educational intervention**

**Journal of Medical Radiation Sciences**

**Authors:**

Olivia Ryan, BMedRadSc (Hon)(RT), ^1,2,3^, Kylie Dundas, MAppSc, BAppSc (MRT)(RT) ^2,3,4^, Yolanda Surjan BMedRadTech (RT), GCertHProm, MHealthSc (ED), PhD ^1^, Doaa Elwadia BAppSc ^2^, Kimberley Nguyen MBBS, FRANZCR ^2, 4, 6^, Michael Cardoso BMedRadPhysAdv (Hons), MBBS ^2,4,5^, and Shivani Kumar MPH, BAppSc (MRS), PhD ^2,3,4^

**Address and Affiliation:**

^1^College of Health, Medicine and Wellbeing, School of Health Sciences, The University of Newcastle, Callaghan, New South Wales, Australia

^2^ Liverpool and Macarthur Cancer Therapy Centres, South Western Sydney Local Health District, Liverpool, New South Wales, Australia

^3^ Ingham Institute of Applied Medical Research, Liverpool, New South Wales, Australia

^4^ South Western Sydney Clinical School, Faculty of Medicine, University of New South Wales, Sydney, New South Wales, Australia

^5^ Centre for Medical Radiation Physics, University of Wollongong, Wollongong, New South Wales, Australia

^6^ Faculty of Medicine, Western Sydney University, Sydney, New South Wales, Australia


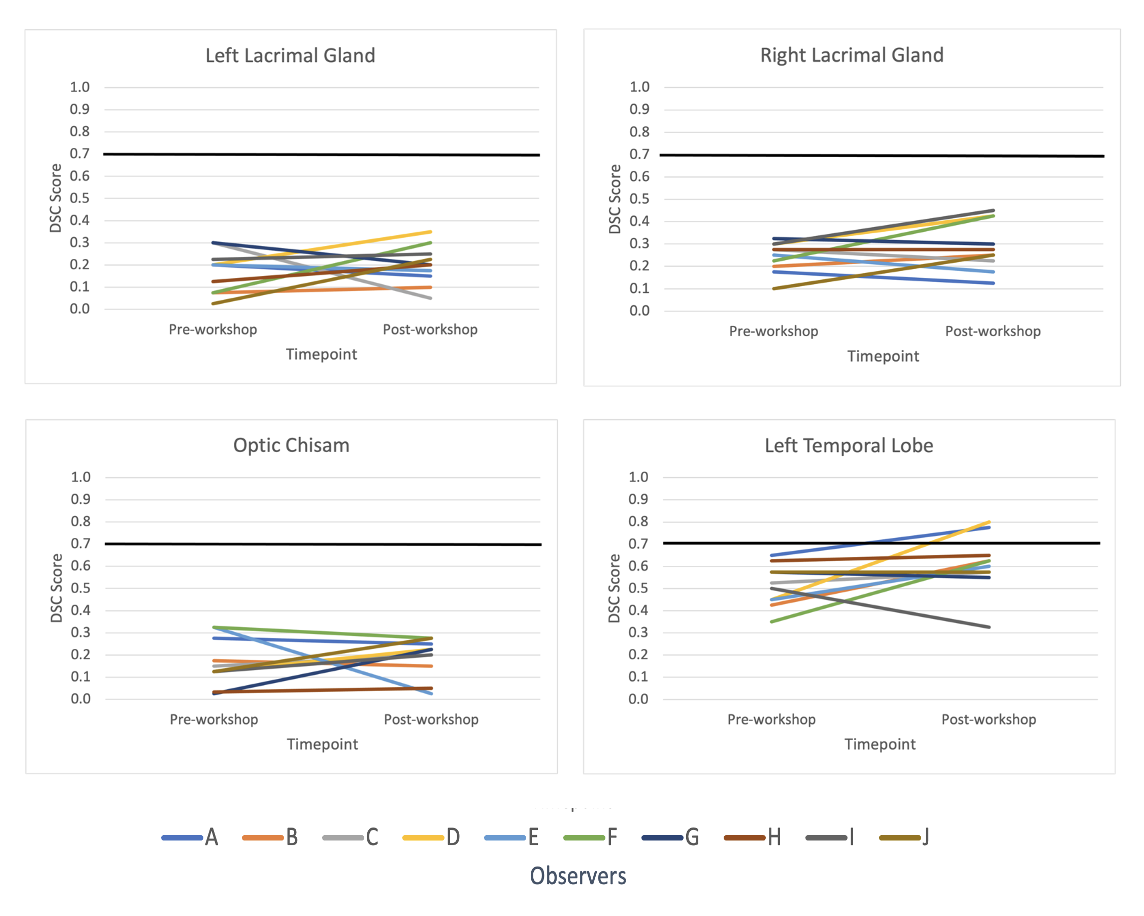
**Corresponding Author Address:** Olivia.ryan@health.nsw.gov.au

**Supporting Information Figure 1.** The organs at risk where >50% of observers showed an increase in mean dice similarity scores but did not achieve a mean DSC >0.7. *DSC* = dice similarity coefficient (DSC >0.7 indicates “good” agreement)

**Supporting Information Figure 2.** The organs at risk where <50% of observers increased in mean dice similarity scores post-workshop. *DSC* = dice similarity coefficient
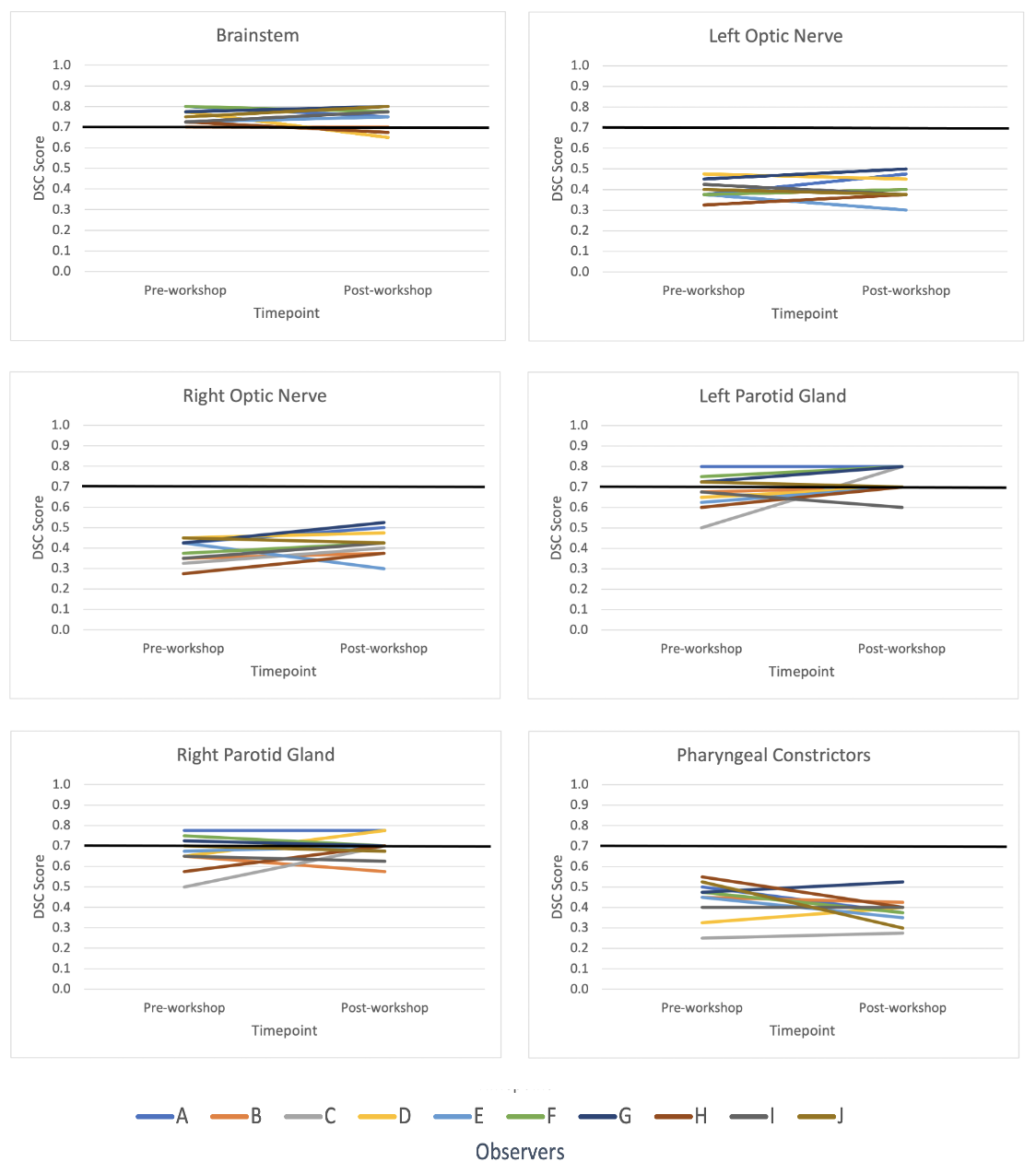

Supplement: Supplementary file 1 — Data S1 Supporting Information [file JMRS-70-59-s001.docx]
